# CCL4L2 is a potential biomarker for differentiating central and peripheral vertigo

**DOI:** 10.3389/fnint.2025.1620845

**Published:** 2025-07-21

**Authors:** Xia Hong, Yuan Li, Chenjuan Tao, Gaofeng Wang

**Affiliations:** ^1^Internal Department One, The Fourth People’s Hospital of Lin‘an District, Hangzhou, Zhejiang, China; ^2^Department of Otolaryngology, Affiliated Hospital of Hangzhou Normal University (Hangzhou Second People’s Hospital), Hangzhou, Zhejiang, China; ^3^Department of Neurology, Affiliated Hospital of Hangzhou Normal University (Hangzhou Second People’s Hospital), Hangzhou, Zhejiang, China; ^4^Department of Otorhinolaryngology, The Fourth People’s Hospital of Lin’an District, Hangzhou, Zhejiang, China

**Keywords:** central vertigo, peripheral vertigo, CCL4L2, biomarker, ROC curve analysis

## Abstract

**Background:**

Central vertigo and peripheral vertigo are common clinical conditions with different underlying pathophysiologies. The identification of reliable biomarkers for differential diagnosis remains a challenge.

**Objectives:**

This study aimed to explore the differential expression of CCL4L2 in the serum of patients with central and peripheral vertigo and assess its diagnostic potential.

**Methods:**

A total of 180 patients (90 central vertigo, 90 peripheral vertigo) were enrolled. RNA sequencing was on serum samples to identify differentially expressed genes (DEGs). Kyoto encyclopedia of genes and genomes (KEGG) pathway enrichment analysis revealed relevant biological pathways. The expression of CCL4L2 was measured using RT-qPCR, and its diagnostic performance was evaluated by Receiver operating characteristic (ROC) curve analysis. The correlation between CCL4L2 expression and biomarkers NSE and S100β was also assessed.

**Results:**

RNA sequencing revealed significant differences in gene expression between central vertigo and peripheral vertigo groups. The KEGG pathway analysis identified several enriched pathways, including NF-κB signaling, where CCL4L2 was a key gene. CCL4L2 expression was significantly higher in the CV group compared to the PV group (*p* < 0.001). ROC analysis demonstrated high diagnostic accuracy for CCL4L2 in distinguishing CV from PV (AUC = 0.909, *p* < 0.001). Additionally, moderate positive correlations were observed between CCL4L2 and NSE (r = 0.475, *p* < 0.001), and a weaker correlation with S100β (r = 0.364, *p* < 0.001).

**Conclusion:**

CCL4L2 may serve as a potential biomarker for differentiating central from peripheral vertigo. Its expression is closely associated with inflammatory pathways, making it a promising target for further investigation in vertigo diagnostics.

## 1 Introduction

Vertigo is a common and debilitating symptom that significantly impacts patients’ quality of life. It can be categorized into central and peripheral vertigo based on the affected anatomical structures (Klokman et al., 2024). Central vertigo arises from lesions in the brainstem, cerebellum, or other central nervous system (CNS) regions, often associated with stroke, multiple sclerosis, or other neurological disorders (Beretti and Desnous, 2023; Choi et al., 2018; Meshref et al., 2022). In contrast, peripheral vertigo is primarily caused by inner ear disorders, such as benign paroxysmal positional vertigo (BPPV), vestibular neuritis, or Ménière’s disease (Djaali et al., 2023; Kim et al., 2021; Lemos and Strupp, 2022). Accurate differentiation between these two types is critical for appropriate management, as their treatments and prognoses differ significantly (Edlow, 2024; Strupp and Brandt, 2008).

Inflammation has been implicated in the pathogenesis of both central and peripheral vertigo (Smith et al., 2025). Central vertigo may involve neuroinflammatory processes, including the activation of microglia and the release of pro-inflammatory cytokines (Gendelman, 2024). Peripheral vertigo, particularly in conditions like vestibular neuritis, is often associated with viral infections and local inflammatory responses (Erbek and Luis, 2024). However, the specific inflammatory pathways and biomarkers involved in these conditions remain unclear. Recent global health events, such as the COVID-19 pandemic, have highlighted the importance of adaptability in research settings and patient care (Drury, 2024).

Recent advances in RNA sequencing technology have enabled comprehensive analysis of gene expression profiles, providing insights into the molecular mechanisms underlying various diseases (Qiu et al., 2024). Recent advancements in understanding physiological markers such as heart rate variability have provided new insights into health and disease (Drury et al., 2019; Laborde et al., 2022). This study aimed to explore the differential gene expression and inflammatory profiles in central and peripheral vertigo patients using RNA sequencing and to identify potential biomarkers for diagnostic differentiation. We focused on the NF-κB signaling pathway, which is a key regulator of inflammation, and investigated the role of CCL4L2, a chemokine identified in this pathway.

## 2 Materials and methods

### 2.1 Patient enrollment

A total of 180 patients who visited our hospital from January 2024 to January 2025 were enrolled in this study, consisting of 90 patients with central vertigo and 90 patients with peripheral vertigo. The patient recruitment adopts a continuous recruitment method, that is, during the study period, all patients who meet the inclusion criteria and are willing to participate in the study are included in the study.

Inclusion Criteria: (1) Patients aged between 18 and 80 years. (2) Diagnosis of central or peripheral vertigo confirmed by clinical evaluation and neuroimaging. (3) Informed consent obtained from all participants.

Exclusion Criteria: (1) Patients with a history of systemic diseases, such as cancer, autoimmune disorders, or chronic infections. (2) Patients with other neurological conditions, including stroke or dementia. (3) Pregnant or breastfeeding women. (4) Patients who had received treatment with immunosuppressive drugs within the last 6 months.

As for central vertigo evaluation, a structured medical history was obtained with particular emphasis on vertigo characteristics, including temporal patterns (episodic frequency, attack duration > 60 s), and concomitant neurological manifestations. The standardized neurological examination protocol incorporated nystagmus analysis, cerebellar testing, and brainstem evaluation. All cases with suspected central etiology underwent neuroimaging prioritization including MRI protocol or CT indications.

As for peripheral vertigo assessment, the diagnostic framework focused on otologic correlates through temporal profile documentation: episodic duration < 60 s with positional provocation (Hallpike-Dix maneuver positivity rate: 72%–86% in BPPV); audiovestibular symptom inventory: Structured questionnaire addressing concurrent tinnitus (67% prevalence in Menière’s), fluctuating hearing thresholds (low-frequency SNHL pattern), and aural pressure (endolymphatic hydrops correlation). The examination protocol included otoscopic evaluation and vestibular function assessments. Specifically, the following tests were performed for vestibular evaluation: Otolith function tests (including cervical and ocular vestibular evoked myogenic potentials), which assess otolith function by eliciting muscle potentials in response to sound or vibration stimuli. Video head impulse test (vHIT), which evaluates semicircular canal function by recording eye movements during rapid head movements. Rotational chair testing, which assesses vestibular function by measuring eye movements in response to rotational stimuli. Computerized dynamic posturography, which evaluates balance function by measuring the patient’s ability to maintain posture under different conditions.

We strictly based on the inclusion and exclusion criteria mentioned earlier. Patients were carefully evaluated to ensure they meet the criteria for central or peripheral vertigo. Patients with other neurological or systemic conditions that may affect the results were excluded.

### 2.2 Serum sample collection and clinical information

Blood samples were collected in the morning after an overnight fast. Approximately 5 mL of blood was drawn from each participant into serum separator tubes. The serum was separated by centrifugation at 1,500 × *g* for 10 min and stored at −80°C until analysis. Clinical information was collected from medical records and provided in the clinical information Table 1.

**TABLE 1:** Clinical characteristics of the subjects.

Groups				
**Characteristics**	**Central vertigo (*n* = 90)**	**Peripheral vertigo (*n* = 90)**	**X^2^/Z/t**	***P*-value**
Age (years)			0.588	0.443
< 55	58	53		
≥ 55	32	37		
Gender	–	–	0.089	0.765
Male	41	43		
Female	49	47		
Symptom duration			6.383	0.094
Seconds	2	5				
Minutes	45	53				
Hours	17	19				
Days	26	13				
Motion sensitivity			154.0	< 0.001**
Yes	83	0		
No	7	90		
History of headaches			3.107	0.078
Yes	34	23		
No	56	67		
Creatinine (μmol/L)	93.40, 29.50	91.67 ± 17.41	−0.289	0.773
BUN (mmol/L)	4.95 ± 0.95	5.17 ± 1.01	1.520	0.130
Albumin (g/L)	43.00, 6.00	43.36 ± 3.72	−0.268	0.788
CRP (mg/L)	19.07 ± 3.23	10.00, 5.00	−11.172	< 0.001**
FPG (mg/dL)	85.00, 5.25	85.00, 5.00	−0.014	0.989
HbA1c (%)	5.00, 0.60	4.97 ± 0.46	−0.293	0.770
WBC (10^9^/L)	14.42 ± 3.67	9.10 ± 1.38	12.88	< 0.001**

**p < 0.01. X^2^, Chi-square analysis; Z, Z-score; T, t-score; BUN, blood urea nitrogen; CRP, C-reactive protein; FPG, fasting plasma glucose; HbA1c, hemoglobin A1c; WBC, white blood cell count.

### 2.3 RNA extraction and quality control

Total RNA was extracted from 200 μL of serum using the miRNeasy Serum/Plasma Kit (Qiagen, Hilden, Germany), following the manufacturer’s instructions. RNA quality and integrity were assessed using the NanoDrop 2000 (Thermo Fisher Scientific, Wilmington, DE, United States), with A260/A280 ratios ranging between 1.8 and 2.1, indicating high-quality RNA suitable for downstream applications.

### 2.4 RNA sequencing

RNA sequencing (RNA-seq) was performed by the Illumina NovaSeq 6000 platform (Illumina, Inc., San Diego, CA, United States). RNA samples from four pairs of central vertigo and peripheral vertigo groups were prepared using the TruSeq Stranded mRNA Library Prep Kit (Illumina), which includes poly-A selection to enrich for mRNA. The libraries were indexed and sequenced in paired-end mode (2 × 150 bp). The sequencing depth was set to approximately 40 million reads per sample.

### 2.5 Data processing

Raw sequencing data were first evaluated for quality using FastQC (v0.11.9). The reads were trimmed and filtered using Cutadapt (v1.18) to remove low-quality bases and adapter sequences. The filtered reads were then aligned to the human reference genome (hg38) using STAR (v2.7.3a). Gene expression levels were quantified using FeatureCounts (v1.6.4). Differentially expressed genes (DEGs) were identified using DESeq2 (v1.30.0), with a threshold of *p* < 0.05.

### 2.6 Kyoto encyclopedia of genes and genomes pathway enrichment analysis

Kyoto encyclopedia of genes and genomes (KEGG) pathway enrichment analysis was performed on the DEGs to identify the biological pathways involved. The KEGG pathway enrichment was carried out using the clusterProfiler package (v3.18.1) in R (Bioconductor). The DEGs were first mapped to KEGG pathways using the KEGG.db package (version 3.10.0). A *p*-value cutoff of < 0.05 was applied to identify significantly enriched pathways. The results were visualized using the dot plot and bar plot functions in clusterProfiler.

### 2.7 RT-qPCR analysis

First-strand complementary DNA (cDNA) was synthesized using the SuperScript IV Reverse Transcriptase (Thermo Fisher Scientific). RT-qPCR was performed to assess the expression of CCL4L2 using specific primers for CCL4L2 (Forward: 5′-GTGGTAGGCAAGCAAGTC-3′, Reverse: 5′-TCAGTTCAGTTCCAGGTCAT-3′), with GAPDH as an internal control. The reaction was carried out using SYBR Green PCR Master Mix (Thermo Fisher Scientific) on a QuantStudio 3 Real-Time PCR System (Thermo Fisher Scientific, Wilmington, DE, United States). Relative gene expression levels were calculated using the 2^–ΔΔCt^ method.

### 2.8 Detection of NSE and S100β

Serum concentrations of the biomarkers NSE and S100β were measured using commercially available ELISA kits. The NSE ELISA Kit (Elabscience, Houston, TX, United States) and S100β ELISA Kit (Elabscience) were used according to the manufacturer’s protocols. The optical density was measured at 450 nm using a microplate reader (BioTek Instruments, Winooski, VT, United States).

### 2.9 Statistical analysis

Data that follows a normal distribution are represented as mean ± standard deviation (SD), *t*-test is used to analyze differences, and Pearson is used to analyze correlations.

Data that do not follow a normal distribution are represented by median M, interquartile range IQR, and analyzed for differences using rank sum test and correlation using Spearman analysis. A correlation coefficient greater than 0.7 indicates a very close relationship; 0.4∼0.7 is closely related; The relationship between 0.2 and 0.4 is average. Statistical significance of the enriched KEGG pathways was determined using the hypergeometric test. A false discovery rate (FDR) correction was applied to adjust *p*-values for multiple comparisons. The analysis was performed using R software (v4.0.2, R Foundation for Statistical Computing, Vienna, Austria). A *p*-value < 0.05 after FDR correction was considered statistically significant. Differences between groups were assessed by Student’s *t*-test, and a *p*-value of < 0.05 was considered statistically significant. Receiver operating characteristic (ROC) curve analysis was performed to assess the diagnostic accuracy of CCL4L2, NSE, and S100β in distinguishing between central and peripheral vertigo. The area under the curve (AUC) was calculated using the MedCalc software (version 20.0). A *p*-value < 0.05 was considered statistically significant. All statistical analyses were conducted using SPSS software (version 25.0, IBM, Armonk, NY, United States).

## 3 Results

### 3.1 Differential gene expression and pathway analysis in central and peripheral vertigo patients

**FIGURE 1:**
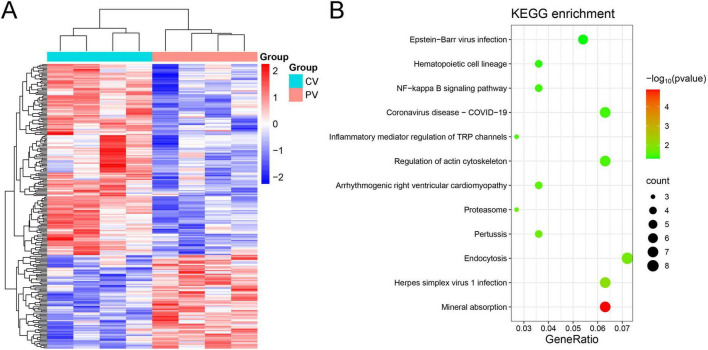
Heatmap and KEGG pathway analysis of differentially expressed genes (DEGs) in central and peripheral vertigo groups. **(A)** Heatmap illustrating the DEGs in central vertigo (CV) and peripheral vertigo (PV) groups after RNA sequencing. The color scale represents the normalized expression levels of genes, with red indicating upregulation and blue indicating downregulation. **(B)** KEGG pathway enrichment analysis of DEGs, highlighting significantly enriched pathways. The size of the circles represents the number of genes involved in each pathway, while the color indicates the significance level of the enrichment (green: highly significant, red: less significant). Notable pathways include NF-κB signaling, inflammation, and viral infections. CCL4L2 is identified as a key gene in the NF-κB signaling pathway.

RNA sequencing was performed on serum samples collected from four patients with central vertigo (CV) and four patients with peripheral vertigo (PV). The heatmap shown in Figure 1A displays the DEGs between the two groups. The results highlight significant variations in gene expression between the CV and PV groups, with distinct clustering of the two groups. Further, KEGG pathway enrichment analysis of the DEGs was conducted, and the results are presented in Figure 1B. The analysis revealed several significantly enriched pathways, including Epstein-Barr virus infection, NF-κB signaling pathway, inflammatory mediator regulation of TRP channels, and actin cytoskeleton regulation. The NF-κB signaling pathway was particularly notable, as CCL4L2, a gene significantly downregulated in the PV group, is implicated in the activation of NF-κB. CCL4L2 likely plays a pro-inflammatory role by recruiting immune cells and promoting the expression of inflammatory cytokines, thereby enhancing the inflammatory response (Lan et al., 2024). Therefore, CCL4L2 was selected for subsequent experiments.

### 3.2 Serum expression of CCL4L2 and its diagnostic value in differentiating central and peripheral vertigo

**FIGURE 2:**
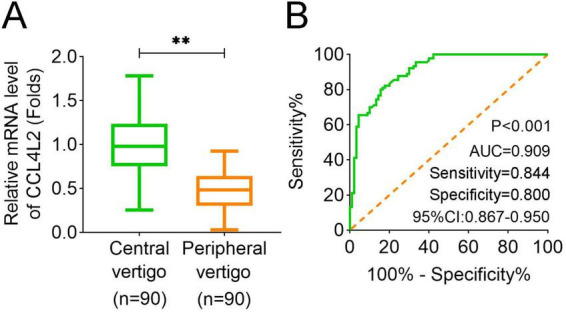
Expression of CCL4L2 in serum and its diagnostic utility for vertigo types. **(A)** Expression levels of CCL4L2 in the serum of central vertigo and peripheral vertigo patients. CCL4L2 expression was significantly higher in the central vertigo (CV) group compared to the peripheral vertigo (PV) group. **(B)** Receiver operating characteristic (ROC) curve analysis of CCL4L2 for distinguishing central vertigo from peripheral vertigo, showing high diagnostic performance with an area under the curve (AUC) of 0.909 (*p* < 0.001, 95% CI: 0.867–0.950, sensitivity value = 0.844, specificity value = 0.800). **p < 0.01.

We measured the expression levels of CCL4L2 in the serum of 90 patients with central vertigo and 90 patients with peripheral vertigo. The results demonstrated that CCL4L2 expression was significantly higher in the central vertigo group compared to the peripheral vertigo group (Figure 2A). Furthermore, the ROC curve analysis revealed that CCL4L2 exhibited a high level of sensitivity and specificity in distinguishing between central and peripheral vertigo (*p* < 0.001, AUC = 0.909, sensitivity value = 0.844, specificity value = 0.800, 95% CI: 0.867–0.950, Figure 2B). The clinical characteristics of 90 patients with central vertigo and 90 patients with peripheral vertigo are summarized in Table 1. The age distribution between the two groups was similar, with no significant difference in the number of patients under and over 55 years (*p* = 0.443). Gender distribution also showed no significant difference (*p* = 0.765). Symptom duration was significantly different between the groups (*p* < 0.001). Patients in the central vertigo group had a higher proportion of symptoms lasting several days, while those in the peripheral vertigo group mostly reported symptoms lasting minutes or hours. A significant difference was observed in motion sensitivity (*p* < 0.001), with the central vertigo group showing higher sensitivity to motion compared to the peripheral vertigo group. There was no significant difference in the history of headaches between the two groups (*p* = 0.078). As for the clinical laboratory markers, significant differences were found in the white blood cell count (WBC, *p* < 0.001), with the central vertigo group showing higher values compared to the peripheral vertigo group. In addition, further multiple linear regression analysis suggested that BUN (*p* = 0.033), CRP (*p* < 0.001), and WBC (*p* < 0.001) were significantly correlated with the expression of CCL4L2 (Table 2).

**TABLE 2:** Multiple linear regression analysis of CCL4L2 expression and related factors.

Analysis parameters							
**Factors**	**B**	**SE**	**β**	**t**	***P*-value**	**95% CI**	
Creatinine (μmol/L)	0.001	0.001	0.071	1.134	0.258	−0.001	0.004
BUN (mmol/L)	−0.051	0.024	−0.131	−2.151	0.033*	−0.097	−0.004
Albumin (g/L)	0.004	0.006	0.044	0.703	0.483	−0.008	0.017
CRP (mg/L)	0.025	0.005	0.379	5.302	< 0.001***	0.016	0.034
FPG (mg/dL)	0.003	0.005	0.039	0.643	0.521	−0.007	0.013
HbA1c (%)	−0.044	0.051	−0.053	−0.867	0.387	−0.144	0.056
WBC (10^9^/L)	0.028	0.007	0.285	3.914	< 0.001***	0.014	0.042

*p < 0.05;

***p < 0.001. B, regression coefficient; SE, standard error; β, standardized regression coefficient; t, t-score; BUN, blood urea nitrogen; CRP, C-reactive protein; FPG, fasting plasma glucose; HbA1c, hemoglobin A1c; WBC, white blood cell count.

### 3.3 Correlation between CCL4L2 expression and biomarkers NSE and S100β

**FIGURE 3:**
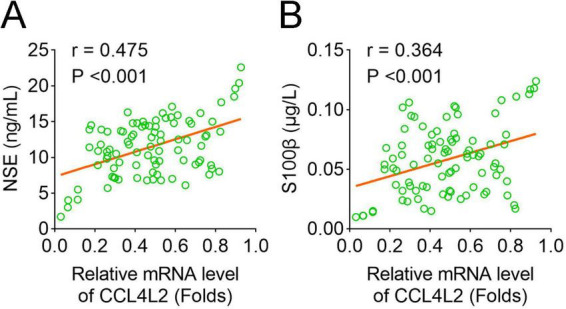
Correlation of CCL4L2 expression with NSE and S100β levels. **(A)** Correlation between the relative mRNA expression of CCL4L2 and serum levels of NSE. A moderate positive correlation was observed (r = 0.475, *p* < 0.001). **(B)** Correlation between the relative mRNA expression of CCL4L2 and serum levels of S100β. A weaker positive correlation was observed (r = 0.364, *p* < 0.001).

The correlation between CCL4L2 expression and NSE levels was evaluated, and the results are shown in Figure 3A. A moderate positive correlation was observed between CCL4L2 mRNA levels and NSE expression (r = 0.475, *p* < 0.001), suggesting that higher CCL4L2 expression is associated with increased NSE levels. Similarly, the relationship between CCL4L2 and S100β expression was assessed, as shown in Figure 3B. A weaker, yet significant positive correlation was found between CCL4L2 and S100β levels (r = 0.364, *p* < 0.001), indicating a similar trend of co-expression.

## 4 Discussion

Our study provides novel insights into the differential inflammatory profiles in central and peripheral vertigo patients. RNA sequencing revealed significant variations in gene expression, with the NF-κB signaling pathway being notably enriched in central vertigo. This pathway is a central regulator of inflammation, orchestrating the expression of numerous pro-inflammatory cytokines and chemokines (Anilkumar and Wright-Jin, 2024; Xu et al., 2019). The identification of CCL4L2 as a key gene in this pathway is particularly noteworthy. CCL4L2, a chemokine involved in immune cell recruitment (Lan et al., 2024; Tsai et al., 2023), was significantly upregulated in central vertigo patients compared to peripheral vertigo patients. This finding suggests that CCL4L2 may play a pro-inflammatory role by promoting the recruitment of immune cells and enhancing the expression of inflammatory cytokines, thereby contributing to the pathogenesis of central vertigo.

The diagnostic utility of CCL4L2 was further supported by ROC curve analysis, which demonstrated high sensitivity and specificity (AUC = 0.909) in distinguishing between central and peripheral vertigo. This indicates that CCL4L2 could serve as a potential biomarker for differentiating these two types of vertigo, which is clinically significant given the challenges in accurate diagnosis. Additionally, the positive correlation between CCL4L2 expression and neuroinflammatory biomarkers NSE and S100β suggests that CCL4L2 may be involved in neuroinflammatory processes (Malau et al., 2024; Ruhnau et al., 2023; Sehr et al., 2019), consistent with its role in the NF-κB signaling pathway. The NF-κB signaling pathway is a key pathway for regulating inflammatory responses, and its activation can promote the expression of various pro-inflammatory cytokines and chemokines, including CCL4L2 (Chen et al., 2018; Mansouri et al., 2024).

In patients with cardiovascular disease, central nervous system lesions may trigger local inflammatory responses, activate microglia and astrocytes, and subsequently activate the NF-κB pathway (Anilkumar and Wright-Jin, 2024). The activation of NF-κB leads to an increase in the expression of pro-inflammatory factors, further exacerbating the inflammatory response (Molaei et al., 2019). The increase of CCL4L2 may exacerbate inflammatory damage by recruiting more immune cells to the lesion site (Lan et al., 2024; Tsai et al., 2023).

Our findings align with previous studies highlighting the involvement of neuroinflammation in central vertigo (Wang et al., 2024). The higher expression of CCL4L2 in central vertigo patients may reflect the activation of CNS immune responses, which are more pronounced compared to peripheral vertigo. This differential inflammatory profile could be attributed to the distinct anatomical and physiological characteristics of the CNS and peripheral vestibular system. The CNS is protected by the blood-brain barrier (BBB), which restricts the entry of peripheral immune cells but can still be activated by local inflammatory signals (Yang et al., 2025). In contrast, peripheral vertigo often involves localized inflammation within the inner ear, which may not trigger a systemic inflammatory response to the same extent. The findings of this study contribute to the growing field of scientific wellness and personalized health approaches as discussed by Hood and Price (Hood, 2023).

However, our study has some limitations. Sample size of RNA-seq is small, it was sufficient to identify DEGs and provide preliminary insights into the molecular mechanisms underlying central vertigo and peripheral vertigo. However, the small sample size does limit the generalization of the gene discovery phase. The clinical sample size, although substantial, may not fully capture the heterogeneity of vertigo patients. Future studies with larger cohorts and diverse populations are needed to validate our findings. Additionally, the functional role of CCL4L2 in vertigo pathophysiology requires further investigation, including *in vivo* and *in vitro* experiments to elucidate its mechanisms of action. It should also be noted that our study lacks a control group, which limits our ability to fully assess the general expression patterns of CCL4L2 in healthy individuals and determine whether the observed differences are specific to vertigo conditions. Future research should incorporate healthy control groups to more thoroughly evaluate the specificity and sensitivity of CCL4L2 as a biomarker.

In conclusion, our study highlights the differential inflammatory profiles in central and peripheral vertigo patients, with CCL4L2 emerging as a promising biomarker for diagnostic differentiation. The findings suggest that CCL4L2 may play a critical role in the neuroinflammatory processes underlying central vertigo. Further research is warranted to explore the clinical utility of CCL4L2 and its potential as a therapeutic target in vertigo management.

## Data Availability

The original contributions presented in this study are included in this article/supplementary material, further inquiries can be directed to the corresponding author.
